# DNA Methylation of the IL-17A Gene Promoter Is Associated with Subclinical Atherosclerosis and Coronary Artery Disease: The Genetics of Atherosclerotic Disease Mexican Study

**DOI:** 10.3390/cimb45120610

**Published:** 2023-12-05

**Authors:** Nonanzit Pérez-Hernández, Rosalinda Posadas-Sánchez, Gilberto Vargas-Alarcón, Óscar Pérez-Méndez, María Luna-Luna, José Manuel Rodríguez-Pérez

**Affiliations:** 1 Department of Molecular Biology, Instituto Nacional de Cardiología Ignacio Chávez, Mexico City 14080, Mexico; unicanona@yahoo.com.mx (N.P.-H.); gvargas63@yahoo.com (G.V.-A.); opmendez@yahoo.com (Ó.P.-M.); mjluna@facmed.unam.mx (M.L.-L.); 2 Department of Endocrinology, Instituto Nacional de Cardiología Ignacio Chávez, Mexico City 14080, Mexico; rossy_posadas_s@yahoo.it

**Keywords:** *interleukin-17A* gene, DNA methylation, subclinical atherosclerosis, coronary artery disease, epigenetics

## Abstract

The interleukin-17 (IL-17) has a crucial role during inflammation and has been associated with cardiovascular diseases, but its role in epigenetics is still poorly understood. Therefore, the aim of this study was to evaluate the DNA methylation status of the *IL-17A* gene promoter to establish whether it may represent a risk factor for subclinical atherosclerosis (SA) or clinical coronary artery disease (CAD). We included 38 patients with premature CAD (pCAD), 48 individuals with SA, and 43 healthy controls. Methylation in the CpG region of the IL-17A gene promoter was assessed via methylation-specific polymerase chain reaction (MSP). Individuals with SA showed increased methylation levels compared to healthy controls and pCAD patients, with *p* < 0.001 for both. Logistic regression analysis showed that high methylation levels represent a significant risk for SA (OR = 5.68, 95% CI = 2.38–14.03, *p* < 0.001). Moreover, low methylation levels of the *IL-17A* gene promoter DNA represent a risk for symptomatic pCAD when compared with SA patients (OR = 0.16, 95% CI = 0.06–0.41, *p* < 0.001). Our data suggest that the increased DNA methylation of the *IL-17A* gene promoter is a risk factor for SA but may be a protection factor for progression from SA to symptomatic CAD.

## 1. Introduction

Coronary artery disease (CAD) is a complex, progressive, and multifactorial pathology influenced by environmental and genetic factors. Currently, CAD represents an important public health problem and is considered the main cause of morbidity and mortality in adult populations worldwide [1,2]. This pathology is the consequence of a chronic inflammatory injury leading to foam-cell and fatty-streak formation, which disrupts normal vascular function. Further, the clinical coronary event occurs after the progression of the inflammatory process and the rupture of atherosclerotic plaque, leading to a thrombotic event with the consequent occlusion of the artery [3,4].

This atherosclerotic plaque frequently becomes calcified, increasing the risk factor of adverse cardiovascular events [5]. Subclinical atherosclerosis (SA) is defined as the presence of coronary calcification without symptoms of cardiovascular disease [6]. This chronic process progresses unnoticed for several years and frequently develops before clinical symptoms appear, such as a coronary event. At this time, coronary artery calcification (CAC) represents a reliable predictor of coronary artery disease (CAD) [7]. Therefore, the CAC score is a powerful tool for rapidly detecting SA in apparently healthy individuals [8,9].

The atherosclerotic plaque formation and the coronary calcification process are highly complex phenomena that involve the participation of different molecules including inflammatory cytokines [10,11,12]. In this regard, interleukin-17 (IL-17) is a family of six cytokine isoforms (IL-17A to IL-17F), IL-17A being its most widely studied member [13]. IL-17A is an important pro-inflammatory cytokine, mainly produced by type T helper-17 (Th17) cells but also by B cells, γδ-T cells, and invariant natural killer T (iNKT) cells [14]. Th17 polarization is motivated by extracellular pathogens, and also by some damage-associated molecular patterns (DAMPs) such as cholesterol crystals [15], is a key feature of fatty streaks and atheroma. Consistently, previous studies have reported an increased IL17-A expression in atherosclerotic lesions, suggesting an active role of this cytokine in the progression of plaque during atherogenesis [16,17]. In addition, increased circulating levels of IL-17A have been reported in several cardiovascular diseases [18,19,20].

A recent in vitro study by Chang et al. demonstrated the effect in human coronary artery smooth muscle cells treated with IL-17A contained in the serum from Kawasaki disease patients which led to the induction of a phenotype that promotes vascular calcification processes [21]. Additionally, a report by Himaritsu-Asano et al. showed that IL-17A promotes vascular calcification in ex vivo cultures of aortas from mice. This calcification was assessed by computed tomography [22]. These findings suggest a key role of IL-17A in vascular calcification, increasing the risk of vascular events in inflammatory processes; however, the precise mechanism has not yet been fully elucidated.

Growing evidence indicates that the initiation and progression of atherosclerotic plaque, including its calcification, may be determined via epigenetic mechanisms; several reports have shown an association between an abnormal DNA methylation profile and CAD [23,24,25,26]. DNA methylation consists of the addition of a methyl group to position 5′ of cytosine in dinucleotide CpG repeats; this process participates in the regulation of gene expression and is the most investigated epigenetic mechanism [26]. In this context, it is likely that *IL-17A* gene methylation is associated with a lower progression of atherosclerotic plaque, but little is known in this field. Therefore, the aim of this study was to establish whether *IL-17A* gene promoter methylation is associated with the incidence of different stages of CAD, i.e., subclinical atherosclerosis and CAD with clinical manifestations.

## 2. Materials and Methods

### 2.1. Design and Subjects

**Figure 1:**
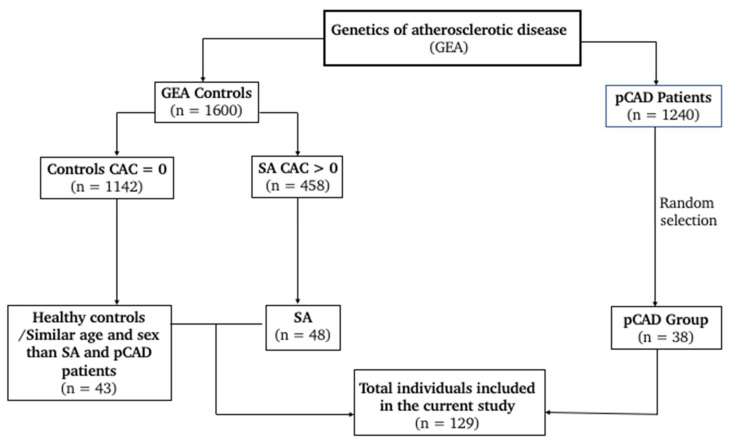
Flowchart for study selection.

This observational and cross-sectional study is nested in the GEA project (Genetics of Atherosclerotic Disease) [27]. We included 129 participants, 38 patients with premature CAD (pCAD), 48 individuals with SA, and 43 healthy controls (Figure 1).

Individuals with SA and controls were apparently healthy volunteers recruited from the open population or blood donors attending our institution. These volunteers did not have a personal or family history or clinical manifestations of CAD. Once they agreed to participate in the study, they were submitted to the computed tomography of the abdomen and chest using a 64-channel multidetector (Siemens, Forchheim, Germany). An expert group of radiologists interpreted the scans and determined the coronary artery calcification (CAC) score through the Agatston method [28].

The control group for this study was constituted of volunteers whose coronary calcium was undetectable (CAC = 0). Moreover, the presence of CAC > 0 was a diagnostic of the presence of coronary lesions [7]; consequently, volunteers with such a condition were classified as patients with SA. Individuals with renal, hepatic, or thyroid disease; oncological diseases; or who were receiving treatment with corticosteroids were excluded. On the other hand, the pCAD diagnostic was made before the age of 55 years in men and before 65 years in women, considering the presence of myocardial infarction, angioplasty, bypass surgery, or coronary stenosis ≥50% determined via angiography. Patients with an acute cardiovascular event within the 3 months prior to the study or with congestive heart failure were excluded. Importantly, CAC score was not determined for symptomatic CAD patients.

Body mass index (BMI) was obtained as weight (kg)/height (m^2^) and biochemical measurements such as plasma glucose, triglycerides, total cholesterol, and high-density lipoproteincholesterol (HDL-cholesterol) were quantified using enzymatic colorimetric procedures (Roche/Hitachi, Germany) on a Hitachi 902 autoanalyzer (Hitachi LTD, Tokyo, Japan). Cholesterol not associated with high-density lipoproteins was obtained by subtracting HDL-cholesterol from total cholesterol [6]. Insulin resistance was estimated using the homeostasis model assessment (HOMA-IR) [29].

All participants in the present study self-reported having Mexican ancestry (of at least three generations). In addition, in order to ensure that population stratification was not a bias in this research, we previously determined the genetic background of this cohort, using at least 265 ancestry informative markers (AIMs) distinguishing African, Amerindian, and European ancestry, and the results of this ancestry analysis revealed a similar genetic background in the AIMs evaluated [30]; therefore, there was no genetic bias in the present study.

The present study was approved by the Ethics and Research committees of the Instituto Nacional de Cardiología (protocol number: 15-947).

### 2.2. DNA Extraction and Sodium Bisulfite Treatment

Genomic DNA was isolated from peripheral blood leukocytes using the QIAamp DNA Mini kit (Qiagen, Hilden, Germany) following the instructions of the manufacturer. Then, DNA purity was evaluated using NanoDrop Spectrophotometer (ThermoFisher Scientific, Waltham, MA, USA) and considered acceptable when the absorption ratio 260 nm/280 nm of the sample was between 1.8 and 2.0. DNA integrity was assessed by 1% agarose gel electrophoresis.

Then, DNA unmethylated cytosines were converted into uracil through the bisulfite method using EpiTect Bisulfite Kit (Qiagen, Hilden, Germany). Methylated cytosines remained unaltered by this method, thus allowing the specific detection of unmethylated or methylated DNA. DNA was denatured at 95 °C for 5 min followed by the conversion reaction at 60 °C during 5 h. After conversion, DNA samples were desulphonated, purified using a preparative column (Qiagen, Hilden, Germany), eluted via centrifugation, and stored at −20 °C until analysis.

### 2.3. Epigenetic Analysis

Methylation in the CpG region of the *IL-17A* gene promoter with sodium bisulfite treatment was performed using methylation-specific polymerase chain reaction (MSP). In brief, 20 ng of DNA, 10 μM of each primer, and 2X Master mix MSP (Qiagen, Hilden, Germany) were used in a final reaction volume of 10 μL. The cycle conditions for *IL-17A* methylated and unmethylated amplicons were as follows: initial activation at 95 °C for 10 min, 40 cycles (95 °C for 15 s, 56 °C for 30 s, 72 °C for 30 s), and 72 °C for 7 min. Each PCR assay included a methylation control, unmethylated control, genomic DNA control (EpiTect PCR Control DNA Set, Qiagen, Hilden, Germany), and a negative control.

The PCR products were analyzed using 2% agarose gel electrophoresis stained with ethidium bromide and then viewed under UV in a transilluminator (UVP Benchtop, Thermo Fisher Scientific, Walthman, MA, USA). We used the MethPrimer software (https://www.urogene.org/methprimer/, accessed on 15 January 2023), to predict and design specific primers to explore a CpG island in the promoter region of *IL-17A* [31]. Primers used to determine the methylated sequences were as follows: Forward 5′TTTTTATGATTTTATTGGGGGC3′ and reverse 5′ATAAACAAAATATAACGCTATCGTC3′ with a product size of 138 bp. The primer for unmethylated sequences was: forward 5′TTTTTTTATGATTTTATTGGGGGT3′ and reverse 5′ATAAACAAAATATAACACTATCATC3′ with a product size of 141 bp. The studied region of *IL-17A* gene relative to transcriptional starting site (TSS) was from −101 bp to +39 bp (141 bp).

### 2.4. Analysis of the Percentage of DNA Methylation

The amplification products corresponding to the MSP assay were separated on an electrophoresis gel as described above. The fluorescence intensity (Fi) of methylated and unmethylated DNA were analyzed using VisionWorks Analysis Software Version 8.20 (Ultra-Violet Products Ltd., Nuffield, Cambridge, UK). The percentage of methylation was calculated as follows:

$$\%\;of\;methylation=\frac{Fi\;methylated\;DNA}{\left(Fi\;methylated\;DNA+Fi\;unmethylated\;DNA\right)}\times 100$$

### 2.5. Statistical Analysis

The quantitative data distribution was determined via the Kolmogorov–Smirnov test. Results were expressed as mean ± standard deviation (SD) or median (interquartile range) for normally and non-normally distributed variables, respectively. Qualitative variables were expressed as frequencies and percentages. To assess the statistical differences between the two study groups, a Mann–Whitney U test was used, and an ANOVA with a Scheffe post-hoc test or a Kruskal–Wallis test for multiple comparisons, depending on the data distribution. A chi-square test was used to compare qualitative variables. For logistic regression analysis, individuals were stratified into two subgroups according to the median of the methylation percentage of the included groups. A first logistic subanalysis included the control subjects and SA patients to determine whether *IL-17A* gene methylation percentages represent a risk of asymptomatic atherosclerosis. In a second model, we included SA and pCAD subjects to statistically establish whether the *IL17-A* gene methylation represents a risk that SA patients will develop symptomatic CAD. The analysis was two-tailed, and the level of significance was less than 0.05 (*p* < 0.05). Statistical analysis was performed in the SPSS v.27 statistical package (SPSS Inc.; Chicago, IL, USA) and GraphPad Prism Software 8.0.1 (GraphPad Software, La Jolla, CA, USA).

## 3. Results

### 3.1. Characteristics of the Studied Population

The main clinical and biochemical characteristics of the study population are depicted in Table 1. We enrolled 38 pCAD patients, 48 individuals with SA, and 43 healthy individuals. The mean age of the pCAD patients and the healthy controls were similar (57.47 ± 6.34 and 56.86 ± 8.85 years old, respectively, *p* > 0.05); however, individuals with SA were older than healthy controls (62.47 ± 8.05 and 56.86 ± 8.85, respectively, *p* = 0.001). In addition, there were more males among the pCAD patients than among the individuals with SA and healthy controls.

### 3.2. DNA Methylation Status of IL-17A Gene Promoter

**Figure 2:**
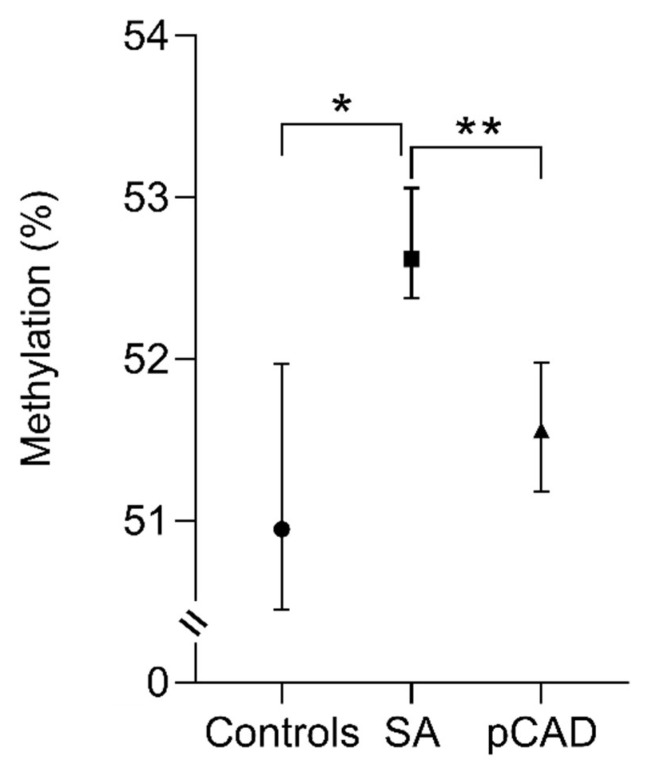
The methylation percentage analysis of the *IL-17A* gene in the study groups. The data are shown as the median value (black circle, square, and triangle) and interquartile range (bars). Kruskal–Wallis and Mann–Whitney U test. * *p* < 0.001, Control vs. SA; ** *p* < 0.001, SA vs. pCAD. SA, subclinical atherosclerosis; pCAD, premature coronary artery disease.

DNA methylation levels were similar in healthy controls and individuals with pCAD. Conversely, individuals with SA showed increased percentages of methylation in comparison with healthy controls and pCAD patients: 52.62 (52.02–53.35) % vs. 50.95 (50.04–52.38) %, and 52.62 (52.02–53.35) % vs. 51.56 (51.04–52.33) %, respectively, with *p* < 0.001 for both (Figure 2).

### 3.3. Association between DNA Methylation Levels of IL-17A Gene Promoter and the Risk of SA and pCAD

The data were stratified into two groups according to the median DNA methylation in the studied population. We first analyzed whether high percentages of methylation were associated with the risk of SA. For this, we performed a logistic regression analysis including control individuals and SA patients; pCAD patients were not considered for this analysis. We defined as reference the group of individuals with methylation levels below the median (52.23%) of the included individuals for this subanalysis. These results revealed that methylation values > 52.23% represent a significant risk of SA (OR = 5.68, 95% CI = 2.38–14.03, *p* < 0.001). We further adjusted the model by sex (model 1), by sex and age (model 2), and by sex, age and glucose levels (model 3). After adjustments, the risk of developing SA with a percentage of *IL-17A* gene methylation above the median of the included individuals in the subanalysis remained statistically significant (Table 2).

In addition, we explored the possibility that SA individuals could progress to symptomatic atherosclerotic (pCAD) in the function of their *IL-17A* gene methylation levels. For this, we included SA and pCAD patients in the logistic regression analysis, using as reference the methylation levels below the median of this subgroup (52.28%). The statistical model revealed a significant OR < 1, indicating that a methylation level > 52.28% represents a protection factor for SA individuals to convert from asymptomatic to symptomatic disease (OR = 0.160, 95% CI = 0.06–0.41, *p* < 0.001). After adjustments by sex (model 1), by sex and age (model 2), and sex, age and glucose levels (model 3) the protection of developing pCAD when SA individuals have a percentage of *IL-17A* gene methylation >52.28% remained statistically significant (*p* < 0.003, Table 3).

## 4. Discussion

Cardiovascular diseases (CVDs) belong to a group of complex pathologies that have an important impact on mortality and, consequently, on public health worldwide. Particularly, CAD has a main inflammatory component that is still not fully understood. In the present study, we evaluated the DNA methylation levels of the *IL-17A* gene promoter to investigate its potential relationship with pCAD and with the potential progression of SA to the symptomatic stage of the disease. Recent reports have considered epigenetic mechanisms in the development of cardiovascular diseases, mainly abnormal patterns of DNA methylation [32], but the knowledge in this field is still scarce.

*IL-17A* epigenetic research is particularly scarce and, as far as we know, this is the first study with an approach related to asymptomatic atherosclerosis. Previous studies have investigated cytokine gene promoter methylation patterns and their relation to CVDs, namely *IL-6* [33,34], a cytokine that is involved in pro-inflammatory processes underlying the development of coronary heart disease. In these studies, the promoter hypomethylation was related to an increased *IL-6* gene expression and an elevated risk of suffering from the disease [33,34]. Similar patterns of hypomethylation have also been reported for CVD-related conditions, such as Behcet’s syndrome with *IL-6* and essential hypertension with IFN [35,36]. These results support the argument that the degree of DNA methylation is inversely associated with gene expression. Consequently, it could be interpreted in this study that the higher the degree of *IL-17A* gene methylation, the lower the gene expression.

IL-17A is a key cytokine produced by a T lymphocyte polarization mainly induced by extracellular stimuli, including antigens and damage-associated molecular patterns (DAMPs) [37]. Previous studies have demonstrated that IL-17A is involved in several pro-inflammatory, prothrombotic, and plaque destabilizing processes on human atherosclerotic lesions [38]. Moreover, considering that cholesterol may act as a DAMP that initiates the inflammatory process in the atherosclerotic lesion, it is expected that it would drive the T cell polarization to the Th17 response [39]. In this context, the methylation of the gene would limit IL-17A expression and, consequently, Th-dependent inflammation activity. Assuming that the severity of atherosclerosis symptoms is associated with the degree of inflammation (active expression of IL-17A), a decreasing percentage of methylation was expected in control subjects, whereas pCAD patients would have the lowest level of methylation, as reported for IL-6 in CAD [33,34]. In addition, SA patients were expected to express an intermediate level of *IL-17A* gene methylation: higher than controls and lower than pCAD patients.

Surprisingly, our data did not show such an increasing gradient of gene methylation from pCAD to controls. Instead, SA individuals had a significantly higher percentage of methylation than controls and pCAD patients. Accepting that SA is a less severe manifestation of the inflammatory process than the symptomatic disease, the higher percentages of gene methylation in SA individuals than in pCAD patients is congruent with the role of IL-17A in coronary artery disease [14]. IL-17A promotes the infiltration of inflammatory cells into the arterial wall and induces TNFα and IL-6, both involved in the atheroma development. Also, IL-17A favors fibrosis, leading to plaque instability [14]. In addition, Th17 cells can also induce the production of other pro-inflammatory cytokines involved in macrophage accumulation in the arterial wall [39]. Therefore, knowing that gene methylation restrains the expression of the protein, it is likely that atheroma in SA individuals does not progress to symptomatic disease because of a lower IL-17A synthesis.

In our study, we found no statistically significant differences in methylation status when comparing healthy controls (CAC = 0) and pCAD patients. It should be considered that atherosclerosis is a complex pathological entity in which several components are involved in the local micro-environment of the subendothelial space. Therefore, we cannot establish that the expression of *IL17-A* is enough to drive the coronary atherosclerosis.

Instead, our results should be interpreted in the context of the pre-existing disease, and they are not extensive to healthy subjects. When the *IL-17A* gene expression is attenuated by an increased methylation in a subject with atherosclerosis, it is likely that the disease progression is retarded. This interpretation is consistent with the mean age of our studied groups; the SA individuals are older than pCAD patients, further supporting the idea of a delay of the appearance of the clinical symptoms due to the higher methylation of the *IL-17A* gene. Further prospective studies are warranted to explore the validity of this hypothesis. Additional epigenetic mechanisms should also be analyzed in asymptomatic individuals.

We recognize some limitations of our work: a prospective interpretation of our results is not possible due to the cross-sectional design of the study. Also, we included only 129 individuals, enough to reach some valid conclusions based on statistical analyses but limited to include more covariables for correction in the logistic regressions. For this reason, pharmacological treatments or lipid profiles were not considered as covariables in statistical analyses. Moreover, we did not measure IL-17A circulation levels in the participants included in this research. Thus, we could not establish whether there were differences in these levels in the study population. Likewise, this limitation prevents us from establishing whether there is an association between the DNA methylation of the *IL-17A* gene studied and the levels of this cytokine.

In summary, our results suggest that increased DNA methylation of the *IL-17A* gene promoter could be associated with SA and with pCAD. However, these findings have not been explored in SA or in coronary disease, so they represent an advancement in the knowledge to establish the basis of epigenetic markers in the early onset of coronary disease. Additional methylation pattern studies of the *IL-17A* gene should be performed to confirm our results.

## 5. Conclusions

We demonstrated an increased *IL-17A* gene methylation in subjects with SA compared to controls and pCAD patients, suggesting a compensatory mechanism to inflammation that retards the onset of symptomatic disease. Furthermore, this study contributes knowledge in the epigenetic field, where there is no previous work on *IL-17A*, so validation of our findings is required.

## Figures and Tables

**Table 1:** Characteristics of the studied groups.

Characteristics	Controls (*n* = 43)	Subclinical Atherosclerosis (*n* = 48)	pCAD (*n* = 38)	* *p*
Age (years)	56.86 ± 8.85	62.47 ± 8.05 **^€^**	57.47 ± 6.34 **^¥^**	0.001
Gender (% male)	15 (34.9)	26 (55.3)	34 (89.5)	<0.001
BMI (kg/m^2^)	25.13 ± 2.94	29.31 ± 3.66 **^€^**	28.60 ± 3.75 **^€^**	<0.001
Glucose (mg/dL)	89 (81–93)	95 (88.5–103) **^€^**	91.50 (84.75–126.75) **^€^**	<0.001
Triglycerides (mg/dL)	101.10 (84–130.20)	157.40 (113.87–236.30) **^€^**	147.40 (111.07–195.42) **^€^**	<0.001
Total cholesterol (mg/dL)	193.80 ± 34.29	194.28 ± 41.44	149.15 ± 35.12 **^€^**^,^ **^¥^**	<0.001
HDL-C (mg/dL)	59 (51–68)	41.79 (35.82–52.20) **^€^**	36.70 (32.82–42.80) **^€^**^,^ **^¥^**	<0.001
Non HDL-C (mg/dL)	132.94 ± 35.20	149.40 ± 42.35	110.58 ± 35.13 **^€^**^,^ **^¥^**	<0.001
HOMA	2.60 (1.85–3.52)	4.78 (3.17–6.96) **^€^**	4.84 (3.81–6.73) **^€^**	<0.001
CAC (Agatston)	0	172.7 (77.47–363.44) **^€^**	ND	<0.001

The data are shown as mean ± standard deviation, median (interquartile range), or number of subjects (percentage). * ANOVA and Post hoc Scheffe, Kruskal–Wallis and Mann–Whitney U test, or Chi^2^ test. **^€^** *p* < 0.05 vs. Control, **^¥^** *p* < 0.05 vs. SA; pCAD, premature coronary artery disease; BMI, body mass index; HDL-C, high density lipoprotein-cholesterol; CAC, coronary artery calcium; ND, not determined.

**Table 2:** Logistic regression models for methylation percentages of *IL-17A* gene association with subclinical atherosclerosis.

Group	Crude Model		Model 1		Model 2		Model 3	
	OR (95% CI)	*p*	OR (95% CI)	*p*	OR (95% CI)	*p*	OR (95% CI)	*p*
1	Reference		Reference		Reference		Reference	
2	5.68 (2.38–14.03)	<0.001	7.74 (2.84–21.08)	<0.001	6.37 (2.34–17.33)	<0.001	6.05 (2.15–16.97)	0.001

The data were stratified into two groups according to the distribution of DNA methylation levels; group 1 included DNA methylation levels lower than the median (52.23%), and group 2 included DNA methylation levels higher than the median methylation. For this subanalysis, only healthy controls and SA patients were included. Group 1 was considered the reference for OR. Model 1 is adjusted by sex; Model 2 is adjusted by sex and age; and Model 3 is adjusted by sex, age, and glucose levels.

**Table 3:** Logistic regression models for methylation percentages of *IL-17A* gene association with symptomatic atherosclerotic.

Group	Crude Model		Model 1		Model 2		Model 3	
	OR (95% CI)	*p*	OR (95% CI)	*p*	OR (95% CI)	*p*	OR (95% CI)	*p*
1	Reference		Reference		Reference		Reference	
2	0.16 (0.06–0.41)	<0.001	0.16 (0.06–0.44)	<0.001	0.19 (0.06–0.57)	0.003	0.19 (0.06–0.57)	0.003

The data were stratified into two groups according to the distribution of DNA methylation levels; group 1 included DNA methylation levels lower than the median methylation (52.28%), and group 2 included DNA methylation levels higher than the median methylation. For this subanalysis, only SA and pCAD patients were included. Group 1 was considered the reference for OR. Model 1 is adjusted by sex; Model 2 is adjusted by sex and age; and Model 3 is adjusted by sex, age, and glucose levels.

## Data Availability

Data supporting the results are available from the corresponding authors upon reasonable request.
